# From Nucleation to Fat Crystal Network: Effects of Stearic–Palmitic Sucrose Ester on Static Crystallization of Palm Oil

**DOI:** 10.3390/foods13091372

**Published:** 2024-04-29

**Authors:** Fien De Witte, Ivana A. Penagos, Davy Van de Walle, Andre G. Skirtach, Koen Dewettinck, Filip Van Bockstaele

**Affiliations:** 1 Food Structure & Function Research Group, Department Food Technology, Safety and Health, Faculty of Bioscience Engineering, Ghent University, Coupure Links 653, 9000 Ghent, Belgium; 2 Nano-Biotechnology Laboratory, Department of Biotechnology, Faculty of Bioscience Engineering, Ghent University, Proeftuinstraat 86, 9000 Ghent, Belgium

**Keywords:** sucrose ester, liquid crystal, palm oil, X-ray scattering, WAXS, SAXS, USAXS, PLM, cryo-SEM

## Abstract

Palm oil (PO), a semi-solid fat at room temperature, is a popular food ingredient. To steer the fat functionality, sucrose esters (SEs) are often used as food additives. Many SEs exist, varying in their hydrophilic-to-lipophilic balance (HLB), making them suitable for various food and non-food applications. In this study, a stearic–palmitic sucrose ester with a moderate HLB (6) was studied. It was found that the SE exhibited a complex thermal behavior consistent with smectic liquid crystals (type A). Small-angle X-ray scattering revealed that the mono- and poly-esters of the SE have different packings, more specifically, double and single chain-length packing. The polymorphism encountered upon crystallization was repeatable during successive heating and cooling cycles. After studying the pure SE, it was added to palm oil, and the crystallization behavior of the mixture was compared to that of pure palm oil. The crystallization conditions were varied by applying cooling at 20 °C/min (fast) and 1 °C/min (slow) to 0 °C, 20 °C or 25 °C. The samples were followed for one hour of isothermal time. Differential scanning calorimetry (DSC) showed that nucleation and polymorphic transitions were accelerated. Wide-angle X-ray scattering (WAXS) unraveled that the α-to-β′ polymorphic transition remained present upon the addition of the SE. SAXS showed that the addition of the SE at 0.5 wt% did not significantly change the double chain-length packing of palm oil, but it decreased the domain size when cooling in a fast manner. Ultra-small-angle X-ray scattering (USAXS) revealed that the addition of the SE created smaller crystal nanoplatelets (CNPs). The microstructure of the fat crystal network was visualized by means of polarized light microscopy (PLM) and cryo-scanning electron microscopy (cryo-SEM). The addition of the SE created a finer and space-filling network without the visibility of separate floc structures.

## 1. Introduction

Palm oil (PO) is obtained from pressing the mesocarp of *Elaeis guineensis*. Compared to other vegetable oils, it is richer in saturated fatty acids. PO is semi-solid at room temperature due to its equal amount of saturated and unsaturated fatty acids [1]. This property makes it a popular ingredient for many food products. Palm oil is kinetically stable and functionalized in food products in the β′ polymorph [2]. Nonetheless, it is prone to recrystallization, where unwanted large crystals are formed, leading to a grainy mouthfeel [3].

Fat crystallization is a complex process including nucleation and crystal growth, resulting in the development of a hierarchical structure [4,5]. The fat crystal network is formed by the self-assembly of the triglyceride (TG) molecules into lamellae. Lamellae stack on top of each other to form crystalline domains, known as crystalline nanoplatelets (CNPs) [6]. CNPs aggregate to larger microstructures that are plate-like, needle-like or spherulitic [7]. In order to steer the fat functionality and make a food product stable over time, additives are often applied. Additives can be used as nucleation seeds, as polymorphic transition retarders or as crystal structure modifiers [8]. Sucrose esters are non-ionic compounds synthesized by the esterification of fatty acids (C8–C22 range) with sucrose [9]. The sucrose molecule has eight possible positions for esterification. Due to these multiple reaction sites, mixtures with tunable degrees of substitution can be created, which results in a complex structural behavior that has barely been studied [10].

Possessing both lipophilic and hydrophilic properties, sucrose esters are suitable emulsifying agents. The amphiphilic nature of sucrose esters is expressed by the hydrophilic-to-lipophilic balance (HLB) and calculated according to a modified version of Griffin’s scale for surfactants: 20 × [weight percent of monoesters in the blend/100] [11]. In practice, the HLB of SEs varies between 1–16 [9,12]. Most researchers apply either very low or very high HLB esters. The advantage of this is that these esters have a high affinity for a certain phase and are, therefore, readily oil or water soluble. Aulton (2002) described that samples with an HLB of 0 to 6 are oil soluble, those with an intermediate HLB (7–9) are water dispersible and those with a high HLB (10–18) are water soluble [13]. Nonetheless, an HLB of 6 is the limit for oil solubility, and some issues with dispersibility might be encountered [14].

SEs have been approved as safe ingredients by the U.S. Food and Drug Administration (FDA), the European Food Safety Authority (EFSA) and the Joint FAO/WHO Expert Committee on Food Additives (JECFA) [9]. SEs are listed in Regulation (EC) No. 1333/2008 as an authorized food additive with the number E473 [15]. The allowed categories of use and the maximum amounts are regulated in the EC Directive 1129/2011 [16]. Commission Regulation (EU) No. 231/2012 restricts the approval to mixtures mainly consisting of mono-, di- and tri-ester compounds by enforcing strict purity criteria [17].

Garbolino et al. (2005) applied 2 wt% of SEs based on laurate (L), palmitate (P) or stearate (S) fatty acids to a palm oil-based blend. In contrast to the laurate ester, the P and S esters ensured a uniform crystalline structure composed of numerous small crystals and eliminated the formation of large granular crystals [8]. Chen et al. (2015) tested SEs (1 wt%) with different acyl chain lengths (L-195, P-170, S-170, O-170 and ER-190) during the crystallization of a palm oil–palm stearin blend. It was found that SEs with saturated and blend-compatible chains (P-170 and S-170) accelerated crystallization and promoted the α-to-β′ transition. In contrast, O-170 had little effect. The FA chains in L-195 and ER-190, dissimilar to the blend, were found to result in the formation of large crystals and delayed the α-to-β′ transition [18]. Tangsanthatkun and Sonwai (2019) studied S170, P170 and L195 in palm olein at concentrations between 0.5–5 wt% and concluded that the effect was dependent on the structure and concentration of the emulsifiers. S and P esters were found to accelerate crystallization via template effects but suppressed later crystal growth. L-195 suppressed the crystallization continuously, probably due to structural incompatibility caused by FA differences [19].

Other studies have been performed, where the effect of SEs on different fat matrices was investigated: a high melting milk-fat fraction [20], blends of a milk-fat fraction and sunflower oil [21], tristearin [22], cocoa butter [23], sunflower stearins [24] and cupuassu fat [25], all at varying concentrations of 0.5–5 wt%. Most recent research focuses on SE applications in whipped cream [26,27,28,29].

It is generally accepted in the scientific field that SEs interact with the fat crystal network via an acyl–acyl interaction, implying that SEs with a similar acyl chain length to the matrix in which they are applied operate as crystal modifiers to enhance crystallization. In contrast, dissimilarity in the acyl chain length might cause the retardation of crystallization [8,18,24]. Additionally, Liu and Binks (2021) discuss the presence of intermolecular and/or intramolecular hydrogen bonds among unsubstituted OH groups and ester carbonyl groups [30]. On top, the large di-saccharide head group might block crystal growth sites [8,18,24]. Although many studies have applied sucrose esters to fat systems, the crystallization of the sucrose ester itself and its effect on the construction of the crystal network have barely been studied. Only Rincon-Cardona (2014) subjected the SE to an X-ray scattering study and concluded that the ester (with a low HLB, and thus, lacking a mono-ester) showed a crystal packing similar to sunflower stearin [24]. The relation of SE addition on the mesoscale of the fat crystal network has been studied by Wakui et al. (2021), who visualized crystal nanoplatelets by means of cryo-TEM and studied the effects of various SEs on the length and width of CNPs [31].

In this study, the effect of a stearic–palmitic SE on the static crystallization of PO is investigated. An SE with a medium HLB of 6 is used, as this ester is approved for applications in food under European legislation, in contrast to some lower HLB esters. A wide toolbox was applied, offering possibilities to study the nano-, meso- and microscale behaviors of the fat crystal network. Different crystallization protocols were applied, mimicking various processing conditions. The samples were cooled in a fast (FC, 20 °C/min) or a slow (SC, 1 °C/min) manner to 0 °C, 20 °C or 25 °C and followed for one hour of isothermal time. The melting and crystallization behaviors were studied by means of differential scanning calorimetry (DSC). The nanoscale structure was studied by means of wide-angle X-ray scattering (WAXS). Simultaneously, ultra-small-angle X-ray scattering (USAXS) measurements were performed, giving indications about the formation of the crystal nanoplatelets (CNPs) and their aggregates. Small-angle X-ray scattering (SAXS) gave information about the chain-length structure and the CNP thickness. Polarized light microscopy (PLM) allowed us to visualize the fat crystal network evolution over time, while cryo-scanning electron microscopy (cryo-SEM) provided more insights in the morphology of the fat crystal flocs.

The scientific relevance of this study lies within the in-depth research on the nanoscale crystallization of an SE with an intermediate HLB. Secondly, the effect of SE addition on the nanoplatelet formation and further aggregation during static palm oil crystallization is of interest. The study highlights the use of USAXS to study the mesoscale of the altered fat crystal network upon SE addition. To the best of our knowledge, this has never been presented. Furthermore, we propose a new sample preparation method for cryo-SEM visualization, enabling complementary mesoscale visualization.

## 2. Materials and Methods

### 2.1. Materials

The sucrose ester (SE) SP30 was kindly provided by Sisterna (Roosendaal, The Netherlands). It was obtained from sugar (beet or cane) and palm oil. Stearic (S) and palmitic (P) acids are the main fatty acids present at approximate concentrations of 70% and 30%, respectively. SP30 contains 37% mono-esters, 34% di-esters, 19% tri-esters and 10% tetra-esters, resulting in a hydrophilic-to-lipophilic balance (HLB) of 6. It appears as a white powder with a melting point of 53–61 °C and caloric content of 7.3 kcal/g. The powder contains 0.9% water, 0.2% free sucrose and <3% free fatty acids.

Palm oil (PO) was kindly provided by Vandemoortele (Izegem, Belgium). The main fatty acids were 45.4 ± 0.1% palmitic acid (P), 38.4 ± 0.1% oleic acid (O), 9.3 ± 0.0% linoleic acid (L), 4.4 ± 0.0% stearic acid (S), 1.2 ± 0.0% myristic acid (M) and 0.3 ± 0.0% lauric acid (La). The solid fat content (SFC) was 48.0 ± 0.1% at 10 °C, 34.2 ± 0.1% at 15 °C, 19.3 ± 0.2% at 20 °C, 10.1 ± 0.0% at 25 °C, 5.4 ± 0.1% at 30 °C and 2.8 ± 0.1% at 35 °C. The triacylglycerol (TG) content of PO is available in De Witte et al.’s study (2024) [32].

The samples studied are PO and PO with the addition of 0.5 wt% of SE, as this is the maximal concentration allowed in most fat-rich food products (not considering infant formulas or soups and broths) under the European legislation EC Regulation 1129/2011-1333/2008. The pure fats are referred to as ‘PO’, whereas the mixture was denoted as ‘POE’, with ‘E’ referring to the addition of the sucrose ester.

### 2.2. Protocols for Crystallization

All samples were preheated to 70 °C for 10 min to erase the crystal memory, then cooled at 20 °C/min (FC) or 1 °C/min (SC) to 0 °C, 20 °C or 25 °C and kept isothermally for one hour. The following abbreviations are used: FC0 = fast cooling to 0 °C, FC20 = fast cooling to 20 °C, FC25 = fast cooling to 25 °C, SC0 = slow cooling to 0 °C, SC20 = slow cooling to 20 °C and SC25 = slow cooling to 25 °C.

### 2.3. Differential Scanning Calorimetry

The crystallization and melting behaviors of the samples were analyzed using differential scanning calorimetry (DSC Q1000, TA Instruments, New Castle, DE, USA). A small amount of sample (±10 mg) was added to aluminum pans (TA Instruments, Zellik, Belgium) and hermetically sealed using a pan crimper press. The instrument was calibrated using indium, azobenzene and undecane. An air-filled pan was used as a reference. The analyses were performed in triplicate. From the resulting profiles, the onset of crystallization and the peak maxima were derived. The melting behavior after one hour of isothermal crystallization was studied by heating all samples at 5 °C/min to 70 °C. The melting profile was characterized by its maxima.

### 2.4. Small-Angle X-ray Scattering

Small-angle X-ray scattering (SAXS) measurements were performed on lab-scale equipment (Xeuss 3.0, Xenocs, Grenoble, France) equipped with a Genix 3D Cu source (wavelength λ = 1.54 Å, Xenocs, Grenoble, France) and an Eiger2R 1M detector (Dectris, Baden, Switzerland). The sample-to-detector distance was 360 mm, resulting in probing a q range of 0.05 Å^−1^ < q < 0.65 Å^−1^. The voltage and current were 50 kV and 0.60 mA, respectively. Quartz capillaries of 1.5 mm in diameter (WJM-Glas, Berlin, Germany) were filled with PO, sealed and put into a Linkam THMS600 heating/cooling stage (Linkam, Redhill, UK) equipped with a liquid nitrogen Dewar and programmed with each of the temperature profiles. A measurement time of 60 s was applied. Due to background corrections and calculation of absolute intensity, each measurement took about 70 s. Every SAXS profile was corrected by subtracting the scattering profile of an empty capillary.

The SAXS data were further processed as previously discussed by De Witte et al. (2024) [32]. The following parameters were obtained:

- The lamellar spacing (d001) was derived from the peak position (q001): d001 = (2 × π)/q001.
- The thickness τ (domain) of the nanoplatelets was derived from the Scherrer equation: τ = (K × λ)/(FWHM001 × cos θ), with K being a numerical constant of 0.9, λ the wavelength, FWHM001 the full width at half maximum from the first-order crystallization peak (radians) and θ the Bragg angle (radians).
- A strain analysis was applied in order to assess the effect of the SE addition on the fat crystal network: FWHM001 × cos θ = (K × λ)/L + 4 × ε × sin θ. FWHM001, θ, K and λ are as described above. L provides a domain size corrected for strain, and ε is the strain. A plot of FWHM001 × cos θ as a function of 4 × sin θ was made based on the first (001)- and third (003)-order SAXS peaks. The slope provided a value for the strain ε. The goal was not to obtain an absolute strain value, but to investigate the strain differences with and without the SE.
- The CNP thickness distribution was derived by means of the Bertaut–Warren–Averbach method (BWA) as described by den Adel et al. (2018) [33] and Rondou et al. (2022) [34].

### 2.5. Wide- and Ultra-Small-Angle X-ray Scattering

Simultaneous wide- and ultra-small-angle X-ray profiles were obtained using the TRUSAXS instrument at the European Synchrotron Radiation Facility (ID02 ESRF, Grenoble, France). The beamline setup is described in detail by Narayanan et al. (2022) [35]. The setup of the experiment is described in detail by De Witte et al. (2024) [32].

### 2.6. Polarized Light Microscopy

A Leica DM2500 device (PLM Leica, Wetzlar, Germany) was used as a polarized light microscope. Applying a polarizer allowed us to distinguish the liquid from the crystalline fat phase and to visualize the fat crystal network on the microscale level. For FC20, FC25, SC20 and SC25, a drop of liquid fat (with or without the ester) was added on a microscopy slide and covered by a cover slip. The temperature protocols specified above were applied using a water-cooled PE120 Peltier system (Linkam, Redhill, UK) attached to the microscope. A 20× magnification lens (type HC PL Fluotar, dry, NA 0.5, WD 3.4 mm) was used. For FC0 and SC0, PO and POE were added to a shear cell CSS450 (Linkam, Redhill, UK) equipped with a liquid nitrogen cooling system. The gap was set at 1000 µm, and no shear was applied. In this case, a 10× lens with a longer working distance (type HC PL Fluotar, dry, NA 0.3, WD 11 mm) was mounted. PLM images were collected in a time-resolved manner by making a video recording.

### 2.7. Cryo-Scanning Electron Microscopy

Cryo-scanning electron microscopy (cryo-SEM) was applied to unravel the 3D morphology of the fat crystal flocs. The samples were crystallized following the protocols described above. Crystallization toward 20 °C or 25 °C was performed on the PE120 Peltier system of the PLM, without cover glass. Crystallization toward 0 °C was performed in a shear cell CSS450 equipped with liquid nitrogen cooling system (Linkam, Redhill, UK). After one hour of isothermal time, the sample (about the volume of a droplet) was transferred to a pre-tempered aluminum cryo-SEM stub covered with carbon tape. The sample was de-oiled by dripping 1 mL of isobutanol on top and letting it evaporate at the crystallization temperature. This was repeated four times. Lastly, 1 mL of acetone was dripped on top and also allowed to evaporate. After vitrification by means of a nitrogen slush, the sample was transferred into the cryo-preparation chamber (PP3010T Cryo-SEM Preparation System, Quorum Technologies, Lewes, UK) under vacuum and conditioned at −140 °C. All samples were sublimated for 45 min at −70 °C, sputter-coated with platinum for 90 s and visualized with a cryo-SEM JEOL JSM 7100F (JEOL Ltd., Tokyo, Japan). The SEM stage had a temperature of −140 °C, and the electron beam had an accelerated voltage of 3 keV.

### 2.8. Graphs and Statistics

Graphs were made using Matlab R2023a. If relevant, statistics were performed with SPSS Statistics 29 (SPSS Inc., Chicago, IL, USA). The normality was verified by means of a Shapiro–Wilk test. A Kruskal–Wallis test was used to compare the means in case of more than two groups present. In the case of a comparison between two groups, a Mann–Whitney U test was applied. A significance level of 5% was selected.

## 3. Results and Discussion

### 3.1. Crystallization Mechanism of Sucrose Ester

#### 3.1.1. Crystallization and Melting Behaviors of the Sucrose Ester

In order to understand the behavior of the SE as a crystal modifier, its crystallization and melting behaviors, crystal packing and thermo-reversibility were investigated. With DSC, the SE powder was crystallized following all temperature protocols applied later on to PO and POE (FC0, FC20, FC25, SC0, SC20 and SC25) and subsequently heated at 5 °C/min. The data can be found in Appendix A. For crystallization, at every condition, four distinct features could be distinguished (Table A1). The crystallization starts at 58–60 °C, with a pronounced crystallization peak around 56–57 °C for fast-cooled samples and around 60 °C for slow-cooled samples. A second peak around 39–40 °C was seen for FC samples and around 43 °C for SC samples. A third feature, a smaller and broader peak, was found around 31 °C and 34 °C, respectively, for FC and SC. A fourth small but steep peak was found in the range of 25–30 °C. Based on previous data, the first crystallization peak can be attributed to the presence of tri- and tetra-esters, and the second peak relates to the stearate mono- and di-ester moieties. The third peak relates to the palmitate moieties [36]. The last peak might be attributed to the presence of a minor fraction in the SE.

Reheating the SE samples resulted in three clear peaks: at 44–45 °C, at 49–50 °C and at 64–66 °C, illustrating that the (majority of the) SE was molten readily when it reached 70 °C (Table A2). The different cooling protocols resulted in similar melting profiles, although for FC20, FC25 and SC25, a small fraction had already melted in the range of 30–40 °C. Previous research has attributed the melting peaks to different fractions: the peak at 49–50 °C can be related to the lower-ester fraction, and its small side peak probably relates to the presence of shorter FA chains. The peak at 64–66 °C relates to the oligo-ester fraction [15,36,37]. According to Szuts et al. (2007), SEs with an HLB of 9–16 undergo a glass transition rather than melting in contrast to more lipophilic esters (HLB 1–3). Microscopy was applied to verify the melting of the ester fraction. It was found that by increasing the temperature, especially from 60 °C to 70 °C, the powder showed spreading, and thus, became fluid and melted, confirming the authors’ conclusions [37].

#### 3.1.2. Subcell Packing and Chain-Length Structure of Sucrose Ester

**Figure 1:**
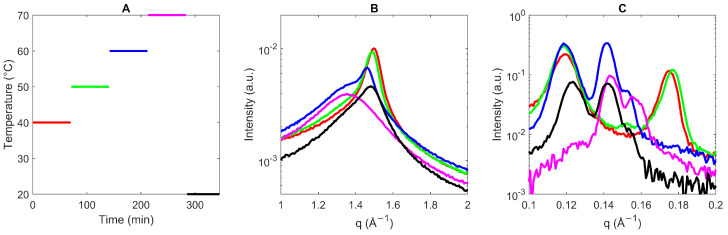
Wide- (**B**) and small-angle (**C**) X-ray scattering (WAXS and SAXS) profiles of sucrose ester SP30 starting from the powdered form as supplied at 40 °C (red), 50 °C (green), 60 °C (blue), 70 °C (pink) and 20 °C (black). The time–temperature profile is illustrated in (**A**). WAXS and SAXS profiles were taken after one hour of isothermal time at the respective temperature.

To study the templating effect of the sucrose ester on PO, the WAXS and SAXS profiles of the pure ester were obtained in order to analyze its time-dependent, solid-state behavior. The WAXS and SAXS measurements were made from 40 °C to 70 °C (±5 °C below and above the melting range), increasing by 10 °C and holding the temperature for one hour (Figure 1, Table A3). Lastly, the sample was cooled to 20 °C.

From the WAXS profiles in Figure 1B, it becomes clear that at 40 and 50 °C, only one peak is present, around q = 1.50 Å^−1^. This value closely relates to the fat peak found for systems with hexagonal packing [10,24]. The spacing of 4.2 Å is rather high in comparison to triacylglycerol (TG)-only systems. It indicates a looser packing with considerable freedom, which might suggest the co-existence of multiple crystal forms [38].

**Figure A2:**
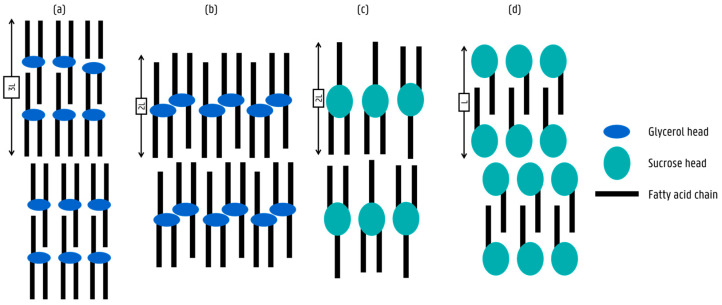
Illustration of different chain-length packings encountered for palm oil and the sucrose ester SP30. (**a**) Triple chain length for triglycerides, (**b**) double chain length for triglycerides, (**c**) double chain length for sucrose ester and (**d**) single chain length for sucrose ester.

In the SAXS region (Figure 1C), at 40 °C, two peaks are present: q = 0.119 and 0.175 Å^−1^, in agreement with spacing of 52.8 and 35.9 Å. For the first-order (001) q = 0.119 Å^−1^ peak, a third-order (003) peak is present at 0.352 Å^−1^, indicative of a lamellar phase. The second-order reflection (002) is absent. Based on the literature, the spacing of 52.8 Å could be related to a double chain-length conformation of mainly tri- and tetra-ester moieties, while the mono- and di-ester moieties could form single chain-length layers with a spacing of 35.9 Å [24]. The single chain length is described by Molinier et al. (2007) [39] as interdigitated bilayers. In parallel, Krog et al. (2007) [40] argued that sorbitan stearate esters with a high mono-ester content pack with a single chain-length configuration, while high amounts of a di-ester and tri-ester form a double chain-length packing. Illustrations hereof can be found in Figure A2.

Heating to 50 °C slightly decreased the intensity at q = 0.177 Å^−1^, which was attributed to the melting of mono-ester moieties (lowest melting point) [15,36]. At 60 °C, the WAXS peak shifted toward q = 1.46 Å^−1^ while being overlayed with a broad peak at 1.35 Å^−1^, consistent with the presence of an amorphous material. This WAXS profile suggested a loosening of the hexagonal conformation. The SAXS analysis illustrates that the mono- and di-ester peaks (q = 0.177 Å^−1^) have disappeared, while a new peak at q = 0.142 Å^−1^ (d = 44.2 Å) appeared. A shoulder at q = 0.152 Å^−1^ was also present, which might be a separation of the palmitate in the thermotropic liquid crystals [41]. Molinier et al. (2006–2007) [10,39] studied the effect of grafting various pure fatty acids (FAs) on sucrose molecules (mono- or di-esters). For the saturated mono- and di-stearate moieties, they found only the SAXS peaks to be persistent up to 110–130 °C. Above this clearing-point temperature, the SE behaves as an isotropic liquid, and the X-ray analysis no longer reveals SAXS nor WAXS peaks. In relation to their study, the peak at q = 0.143 Å^−1^ was also found for di-esters, while the peak at 0.160 Å^−1^ was also found for mono-esters. Amphiphilic molecules with cyclic or sugar polar heads exhibit, in most cases, a smectic A* phase (SmA*) [10]. This phase behavior depends on, amongst other things, the volume ratio of the FA to the polar head group. A smectic phase is a type of ordered phase where the order is only present in one direction (e.g., layering). The A type shows no tilt, whereas the C type shows tilted molecules along the normal layer. The asterisk refers to the fact that the molecules are chiral, and their symmetry is thus reduced [39].

**Figure A3:**
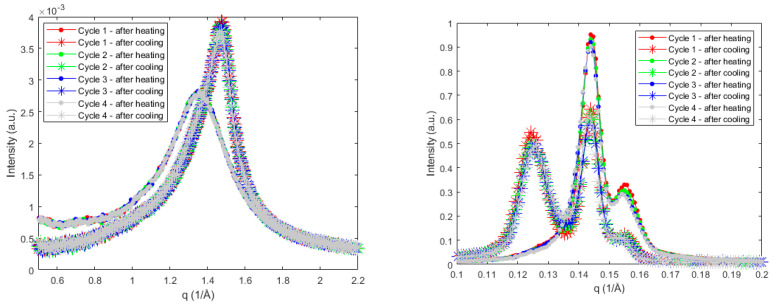
Assessment of thermo-reversibility of sucrose ester crystallization. WAXS (**left**) and SAXS (**right**) profiles found upon thermal cycling between 70 °C (‘after heating’) and 20 °C (‘after cooling’).

Finally, cooling to 20 °C resulted in the presence of a hexagonal (WAXS peak at q = 1.48 Å^−1^) and lamellar phase (SAXS peaks at q = 0.124 and 0.142 Å^−1^, with the presence of higher-order peaks). It must be stressed that the profile with the initial peaks at 40 °C is not obtained again after cooling to 20 °C. This unraveled that the profile of the ester powder is not similar to the ester that has been melted at least once, probably due to mixing during melting [37]. Considering this important finding for the processing of food samples and preparation of mixtures for analysis, the reversibility of the structural changes was assessed. Nonetheless, a further analysis confirmed that upon repeated heating to 70 °C and cooling to 20 °C, similar peaks were continuously obtained (Figure A3).

### 3.2. Effect of Sucrose Ester Addition on Crystallization of Palm Oil

#### 3.2.1. Onset of Crystallization

**Figure A4:**
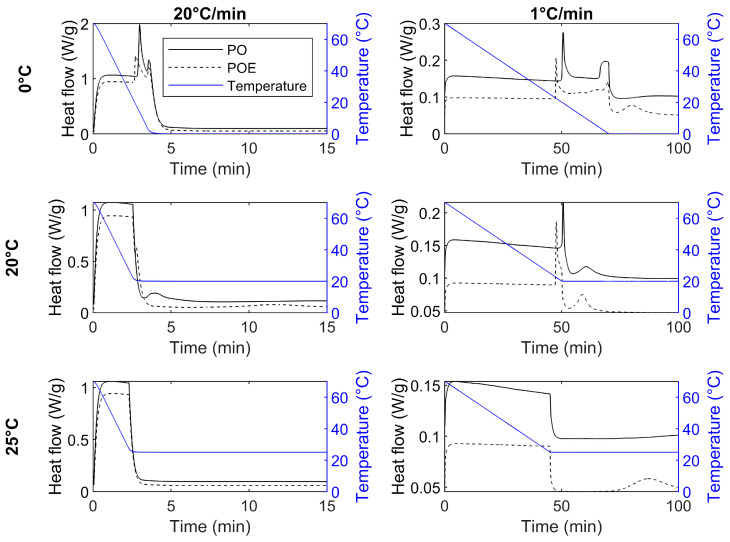
Thermograms obtained using differential scanning calorimetry (DSC) for comparison of PO and POE crystallization upon application of the different cooling protocols. The time range shown is limited for readability.

The crystallization behaviors of PO and PO with the addition of 0.5 wt% of SE (POE) were assessed by means of differential scanning calorimetry (DSC) under fast and slow cooling to different end temperatures: 0, 20 and 25 °C. The results are shown in Appendix B. The crystallization and melting of PO and POE are shown in Figure A4 and Table A4. For FC0, crystallization started for PO and POE before 0 °C was reached, at 17.5 and 20.6 °C, respectively. During cooling, two peaks were found: at 13.8 °C and 2.3 °C for PO and at 18.6 °C and 2.4 °C for POE, indicating, in both cases, the crystallization of two fractions (high and low melting). Both fractions crystallized sooner upon the addition of the SE. Later, in the isothermal phase around 29.4 min for PO and sooner, at 12.2 min, for POE, a broad bump, consistent with a polymorphic transition, was found.

In the case of slow cooling (SC0), sharp crystallization peaks were found around 60 min and 20 °C. POE crystallization started again before PO crystallization. A second peak was formed around 68.6 min for PO, when an isothermal temperature of 0 °C was reached. For POE, this peak seemed to be split in two fractions (65.5 and 70.0 min). During the isothermal phase, a peak at 80.5 min was found for POE, while for PO, a broad bump situated at 93.5 min was seen, equally indicating a phase transition. Domingues (2022) [38] studied the addition of 1% S-370 to PO and applied an intermediate cooling rate of 10 °C/min. Their study showed that mainly the low melting fraction was impacted. Chen et al. (2007) [18], on the contrary, also reported a minor influence on the low-melting fraction when adding 1% S-170 or P-170 to a palm fat blend.

At 20 °C, only the high melting fraction of palm oil crystallizes. Both the crystallization peak and polymorphic transition peak were found sooner upon the addition of the SE. At 25 °C (FC25 and SC25), it was often difficult to detect crystallization due to the low SFC (around 10%). This might indicate that a larger fraction crystallizes for POE or that the crystallization speed is higher for POE and more fat crystallized concurrently, making it detectable using the DSC device.

In general, the SE addition was found to speed up crystallization and produce narrower and more intense peaks, even at the low concentration studied. No crystallization peaks were found in the high-temperature range, which could be attributed to the crystallization of the SE itself. The SE can, therefore, act as a seed for crystallization or can cocrystallize with the fat. Several authors describe the seeding mechanism for low HLB esters, in which the crystallization peak is situated at higher temperatures compared to that of the fat matrix [24,38,42]. However, as the SE studied has two crystallization peaks, both phenomena might be applicable.

#### 3.2.2. Polymorphic Transition and Lamellar Thickness

##### Polymorphism

**Figure 2:**
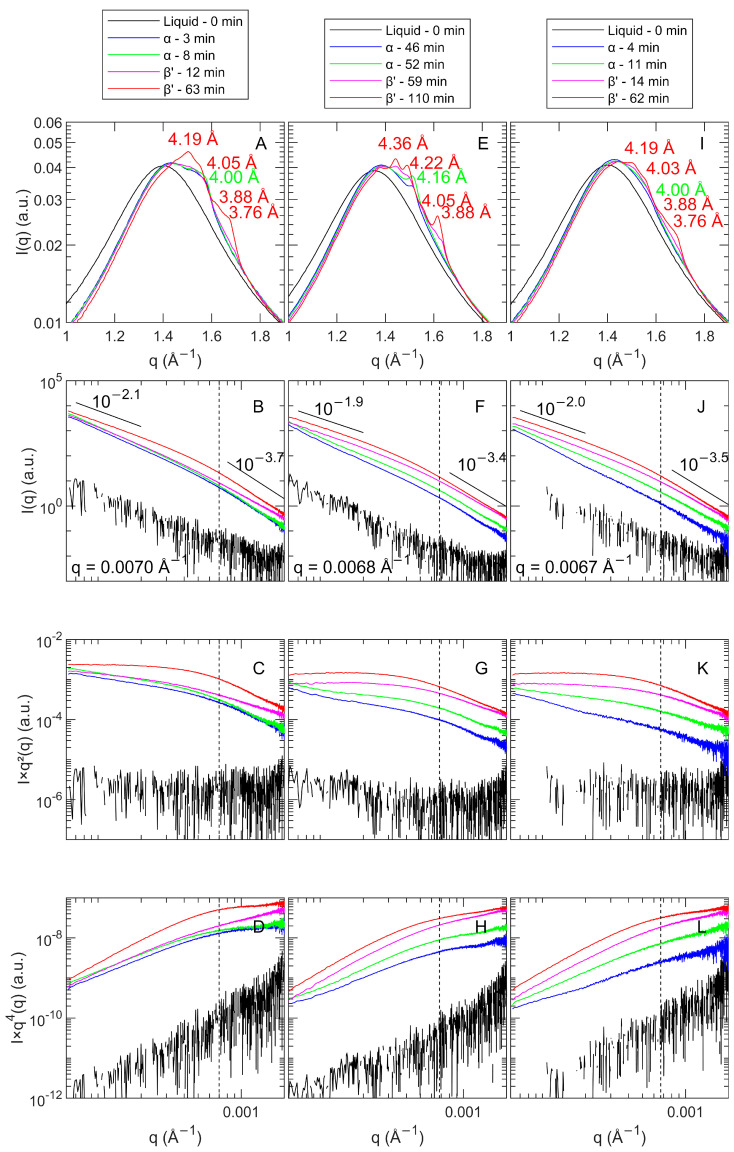
Selected wide- and ultra-small-angle X-ray scattering (WAXS and USAXS) profiles of POE crystallized at FC20 (**A**–**D**), SC20 (**E**–**H**) and FC25 (**I**–**L**).

A simultaneous synchrotron wide- and ultra-small-angle X-ray scattering (WAXS and USAXS) setup allowed us to study the fat crystallization on the nano- and mesoscale in situ. For pure PO, De Witte et al. (2024) concluded that at the nanoscale, similar polymorphic transitions (α to β′) were encountered for FC20, SC20, FC25 and SC25. From the presented WAXS profiles (Figure 2A,E,I), it is clear that the same polymorphic pathway was found for POE samples and that all samples predominantly showed the β′ polymorph after one hour of isothermal crystallization. As described before, the SE shows a hexagonal packing; thus, crystallization via the α polymorph was expected. The start of crystallization appeared earlier with the addition of SE, and the polymorphic transition was quickened. The same conclusions were derived earlier from DSC crystallization data. The transformation to β′ is promoted because of the loose α (hexagonal) confirmation formed by the sucrose head groups [18]. Similar findings were reported by Tangsanthatkun and Sonwai (2019) [19]. It can be concluded that the addition of a low amount of SE (0.5 wt%) did not influence the polymorphic habit of PO.

##### Lamellar Spacing

The small-angle X-ray scattering results for crystallization at 0 °C can be found in Appendix C (Figure A6). As long as the samples are liquid, a peak at q = 0.140 Å^−1^ is present, resulting from the SE mesophase. When PO is fast-cooled and crystallized at 0 °C, a peak around q = 0.136 Å^−1^ (d = 46.2 Å) consistent with a 2L chain-length structure was found [43]. About two minutes later, an extra peak appeared around q = 0.120 Å^−1^ (d = 52.4 Å). This peak was described by Sainlaud et al. (2022) [1] with the term ‘φ phase’. The authors described this phase as temporary and present before the formation of the triple chain-length structure (3L). Although the peak around q = 0.136 Å^−1^ evolved over time to overlap with another peak around q = 0.152 Å^−1^ (d = 41.3 Å), no evidence of the 3L chain-length structure was found within the time frame of the experiment. The end profile (Figure 2A, red) results from an unfinished α-2L-to-β′-2L transition. For SC0, the phases encountered resembled those of FC0.

For POE in FC0, the same phases were formed as for PO. The end profile (Figure 2C, red), however, showed that less α-2L was preserved compared to PO. Under slow cooling conditions, the formation of the φ phase was clearly moderated, as after a few minutes, this phase already disappeared in POE, in contrast to PO. Moreover, the crystallization was sooner directed toward β′-2L, and less α-2L remained present after one hour of isothermal crystallization.

**Figure 3:**
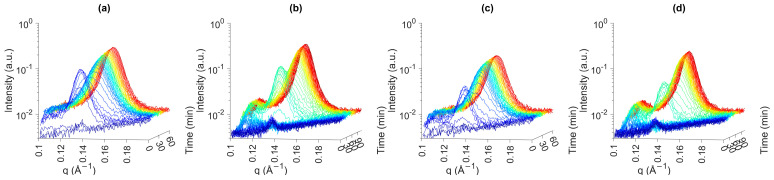
Small-angle X-ray scattering (SAXS) profiles for time-resolved crystallization of POE. (**a**) FC20, (**b**) SC20, (**c**) FC25 and (**d**) SC25. Colors blue to red indicate the evolution over time.

At 20 °C (fast and slow cooling), a polymorphic transition from α-2L to β′-2L was found, with and without the presence of SE (Figure 3). The addition of SE seemed to shorten the transition phase, but the overall profiles for PO and POE were very comparable. It is hypothesized that the SE serves as a seed to bring TG molecules together, which then quickly transform into a more stable subcell [18]. Other authors state that additives might cause structural lattice defects (voids), ensuring more mobility for TGs, which enhances polymorphic transitions into more stable subcells [44].

At 25 °C, both for FC and SC, the addition of 0.5 wt% of SE accelerated the crystallization. Nonetheless, the presence of SE resulted in a shorter life span of the α-2L polymorph and a faster transition towards the final β′-2L polymorph. All d001 spacings were found to be around 42 Å after one hour of crystallization (Table 1). For the samples containing the SE, a small peak around q = 0.139 Å^−1^ (d = 45.2 Å) is consistent with the SE peaks discussed earlier.

#### 3.2.3. CNP Structure and Aggregation

##### Crystal Nanoplatelet Thickness and Thickness Distribution

The Scherrer equation or the Bertaut–Warren–Averbach (BWA) method can be used to assess the crystal nanoplatelet (CNP) thickness [32,34]. Due to incomplete polymorphic transitions, the measurements at 0 °C were excluded from the calculations. For the samples crystallized at 20 °C or 25 °C, the results can be found in Table 1. In comparison to PO, the application of different cooling protocols had a more pronounced effect on the crystallization of POE, as larger differences in CNP thicknesses were found. By applying the Scherrer equation, values ranging from 9.6 (FC20) to 12.3 (SC25) were found. For BWA, a range between 8.2 (FC20) and 10.3 (SC25) clearly revealed the same trends. As such, in contrast to PO, the CNP thickness in POE samples was decreased by applying a higher cooling rate.

**Figure 4:**
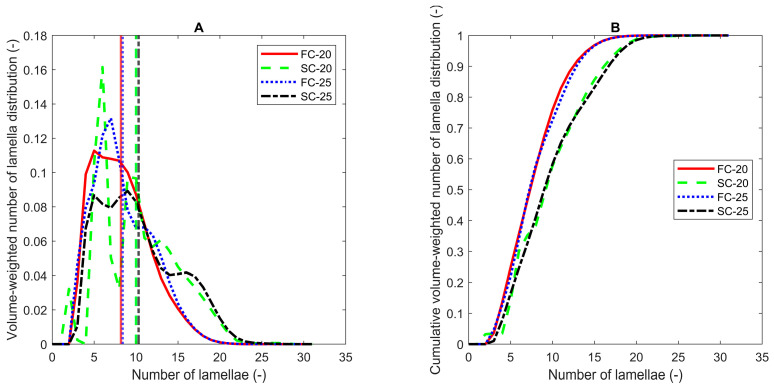
Volume-weighted crystallite thickness distribution curves (**A**) and their cumulative counterparts (**B**) for POE as a function of the number of lamellae obtained using the BWA method after one hour of isothermal crystallization. Vertical lines indicate the volume-weighted mean number of lamellae.

The volume-weighted crystallite thickness distributions and their cumulative counterparts obtained by the application of the BWA method are shown in Figure 4. The figure illustrates that two groups could be distinguished. On the one hand, for POE, FC20 and FC25, a bell-shaped curve skewed to the right was found. On the other hand, for POE, SC20 and SC25, the curve seemed to consist of three fractions around 5, 10 and 18 lamellae.

In all cases, the Scherrer and BWA methods demonstrated that the thickness of the CNPs decreased upon the application of the SE. Precaution must be taken when interpreting these data. The Scherrer equation and the Bertaut–Warren–Averbach method (BWA) rely on the SAXS peak shape. However, this shape is determined not only by the crystal size, but also by instrumental peak broadening and crystal strain. In this study, the authors discern the presence of instrumental peak broadening, but as the values obtained from SAXS are only compared to values obtained on the same device, no peak broadening correction was made.

##### Crystal Strain

Crystal strain is a distortion of the fat crystal particles by the presence of foreign (non-TG) molecules [45]. In the case of the addition of the SE to PO, the assessment of strain is insurmountable, as the SE molecules might change the structure of TG lamellae. The results are shown in Appendix D. The crystal strain assessments of PO and POE (Table A6) showed no significant differences in the strain values found for PO and POE. Moreover, it is clear that the strain differences found under fast and slow cooling are similar to the strain differences found for the addition of the SE. As such, we conclude cautiously that the strain has not affected the outcomes of the Scherrer and BWA analysis.

##### Crystal Nanoplatelet Size

With respect to the CNPs, De Witte et al. (2024) [32] concluded, based on USAXS data, that for PO, a faster cooling rate and a lower isothermal temperature ensured the formation of smaller crystal nanoplatelets (CNPs). For FC25 and SC25, no differences were detected. Following this, only FC20, SC20 and FC25 were studied for POE. Figure 2 presents the WAXS and USAXS results for POE. Table 2 provides a clarifying overview of all USAXS results obtained for PO and POE samples.

The POE USAXS profiles showed less pronounced changes upon the polymorphic transitions compared to pure PO. It was assumed that as the SE increased the speed of crystallization, there was less time to build a well-organized fat crystal network [8]. The slopes at low q were found to be 2.1, 1.9 and 2.0 for FC20, SC20 and FC25, respectively. This zone can be seen as a horizontal zone in the Kratky plot (Figure 2C,G,K), which is indicative of a flat, lamellar-based shape, similar to what was found for PO [32,46]. The slopes at high q are in the same range (3–4) as for PO, and thus, illustrate a similar interface contrast between the liquid and the solid fat [32].

The bending section interconnecting the straight sections at high and low q was found to be situated at 0.0070 Å^−1^, 0.0068 Å^−1^ and 0.0067 Å^−1^ for, respectively, FC20, SC20 and FC25. The differences between FC and SC protocols are less pronounced than those for CNP thickness. Although the cut-off values theoretically show the same trend as found for PO, with lower cut-off points for higher cooling rates and lower isothermal temperatures, the differences between the absolute values are minor and probably indicate that a limiting CNP size was encountered upon the addition of SE. In any case, the cut-off points indicate that the CNP size with the SE addition is much smaller than that for pure PO.

In addition, PO and POE were quickly crystallized to 0 °C. The results are illustrated in Appendix C (Figure A7) and Table 2. The USAXS profiles seemed to saturate. The middle and high q range after 4 min is similar to that found at the end of the cooling period (3 min 30 s). From 4 to 15 min, only the low q region seemed to slightly further increase in intensity until almost a straight profile was found. This profile indicates the saturation of the USAXS technique, as an electron density contrast is no longer present. In the case of fat crystallization, this means that the solid fat content has increased to over 50% and that the crystal network can no longer be discriminated from the oil phase. After 15 min, the experiment was stopped, as no further information was obtained. We should thus conclude that USAXS is a method well suited for the study of low solid fat samples. A similar distinction between the interpretation at high and low solid contents was made in the past by Peyronel et al. [47,48]. Despite the fact that the samples could not be studied after 1 h of isothermal time, it is clear that the initially formed CNPs are smaller in POE than in PO, with cut-off points of 0.0066 Å^−1^ and 0.0054 Å^−1^, respectively.

**Figure 5:**
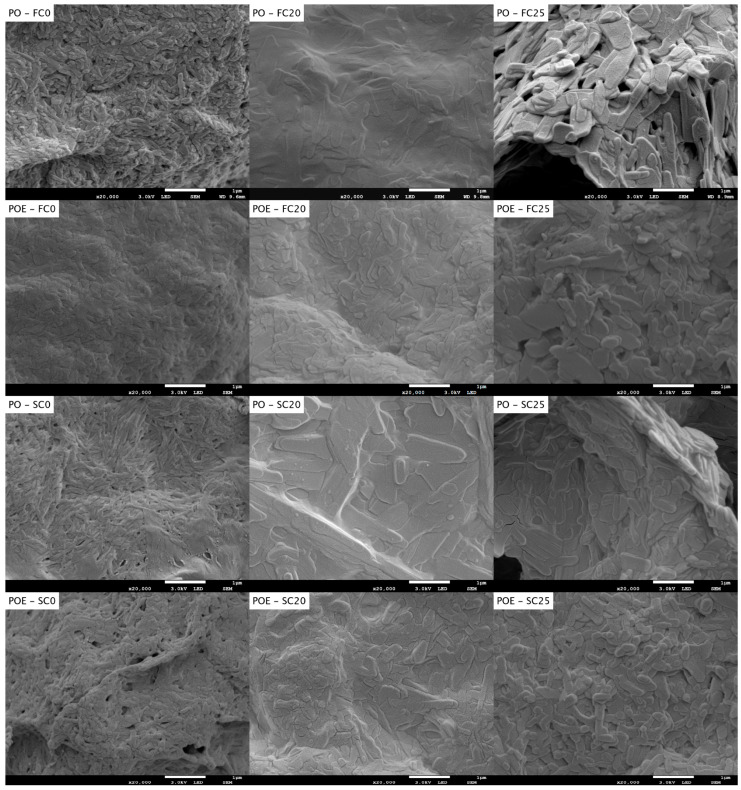
Cryo-scanning electron microscopy images of PO and POE showing CNPs and their aggregates (magnification 20,000×; scale-bar = 1 µm), crystallized following FC0, FC20, FC25, SC0, SC20 and SC25 and kept one hour isothermally before de-oiling.

In order to validate the data obtained using USAXS, the samples were studied using cryo-SEM (Figure 5). The visualization of the solid fat crystal network by means of SEM requires the remaining liquid oil to be removed from the sample. To ensure as minimal damage to the network as possible, a modification to the procedure, as reported in our previous study, was made [32]. The samples were no longer added to an excess of solvent, then de-oiled and later moved to the sample holder for visualization. Instead, the sample was first transferred to the holder, and then, only the top layer was de-oiled by dripping solvent on top. This modification allowed us to maintain the fat, to a larger extent, in its original confirmation.

Upon 20,000× magnification, the separate building blocks (CNPs) of the fat crystal network could be seen. The resolution of the images was improved using the current de-oiling method. The images reveal that the CNPs formed for PO, quickly cooled to 0 °C, are clearly smaller and more uniform in size compared to those at a higher temperature (20 and 25 °C).

Although cryo-SEM is not an ideal technique to make conclusions in terms of sizes, the visualizations equally suggested that the CNPs in POE samples are smaller than in PO samples. The same conclusion was drawn from the USAXS results. Wakui (2021) [31] studied, using cryo-TEM, the CNP length in palm-based water-in-oil emulsions without and with added sucrose esters (0.5%), with different substituting fatty acid chains. The authors concluded that upon the addition of palmitic or stearic esters with an HLB of 1, the CNPs became shorter. The stearic ester equally decreased the CNP width (measured using XRD), which was not the case for its palmitic counterpart. Two hypotheses could be made: (1) including the SE molecules in the CNPs might block growing sites, and thus, hinder further CNP growth and (2) adding the SE to the sample created many nucleation sites simultaneously, creating smaller but numerous CNPs.

##### Microstructure of Fat Crystal Network

**Figure 6:**
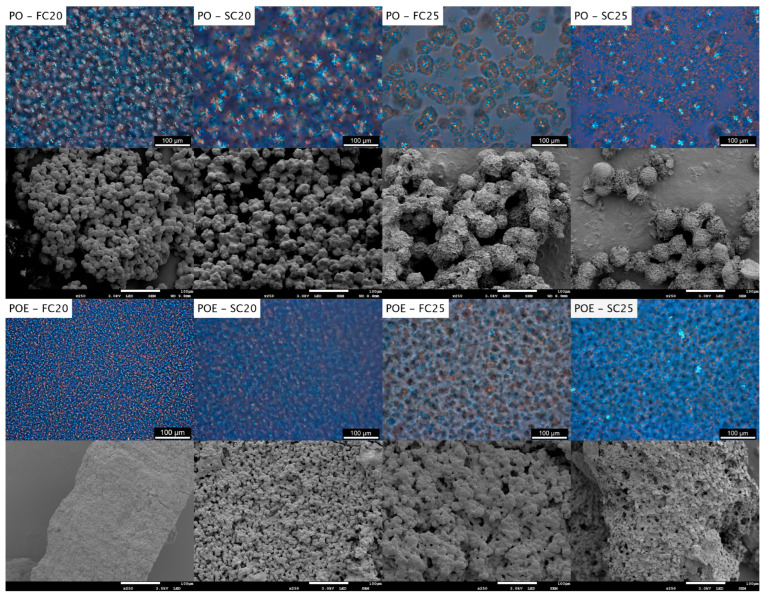
Polarized light microscopy (PLM) images of PO (**first row**) and POE (**third row**) and their cryo-SEM counterparts (**second** and **fourth row**) crystallized following FC20, SC20, FC25 and SC25 and kept isothermally for one hour. Scale bar for techniques represents 100 µm.

Polarized light microscopy is a commonly used technique to visualize the microstructure of the fat crystal network. The networks of FC20, FC25, SC20 and SC25 were visualized using a Linkam Peltier system. However, since this system did not allow for cooling to 0 °C, a nitrogen-cooled stage was used to create the images at 0 °C (Appendix C, Figure A8). PO forms very distinct networks for the different cooling protocols [32]. The finest network structure was found for FC20. Nonetheless, Figure 6 shows that upon the addition of the sucrose ester, the network became finer and homogeneous. A similar finding holds for SC20, where the network of POE seemed coarser than the one ofFC20 but was still finer than the network encountered for pure PO under the same conditions.

At 25 °C, PO has the tendency of forming dense centers during crystallization in the α polymorph. Later on, a fanning crown, crystallizing in the β′ polymorph, surrounds the center [32]. In contrast to PO, the networks found for POE at FC25 and SC25 resembled the one found for SC20, where no separate crystal flocs and crystal floc centers could be distinguished from each other.

Other studies, which applied several SEs at varying temperatures, equally concluded that the addition of SEs affected the size and morphology of the fat crystals and that a more homogeneous and compact crystal network was formed [8,18,31,49]. From this, it can be concluded that the SE added enhanced PO nucleation and suppressed further crystal growth [19]. Referring to Figure A8, the same conclusion can be drawn at 0 °C. Several authors have discussed the importance of FA similarity between the SE and the fat matrix in order to efficiently modify the crystallization behavior [8,18]. As the SE studied is highly compatible with PO, it is likely that the alkyl chains of the SE moieties were incorporated into the alkyl chains of the growing PO crystals, and by that action, blocked crystal growth. It remains, however, debatable to which extent TGs and SEs can effectively cocrystallize [8,19]. Chen et al. (2015) reported that after the rapid start of crystallization caused by SE addition, the viscosity of the sample increased, leading to reduced mobility, and hence, limited crystal growth. A last possibility is that during rapid crystallization, TGs have too little time to organize themselves into a well-ordered and large crystal structure [18].

In order to further unravel the crystal floc morphology, PLM images were compared to cryo-scanning electron microscopy images at a similar magnification (Figure 5). For pure PO, the method applied resulted in images comparable to those found and discussed in our previous research paper, which validated the applied method [32]. Similar to the PLM images shown, it is clear that FC25 has bigger flocs compared to FC0 and FC20.

For the POE samples, it is clear from Figure 5 and Appendix C that the floc substructure of the fat crystal network became invisible for FC0 and FC20. This might be due to the many nucleation sites appearing in the presence of the SE or the fast crystallization kinetics of the POE samples. For FC25, flocs are visible but are much smaller than for PO. Regardless of its cause, the addition of the SE tempered the crystal growth in terms of the floc size. Similar modifications to the fat crystal network upon the addition of the SE and SE/lecithin mixtures were seen by Bin Sintang et al. (2017) [50].

In the case of slow cooling, the floc structure of POE remained visible at all temperatures. The images confirmed the smaller floc size for POE compared to PO as found using PLM. Cryo-SEM visualization provided a distinction between samples with a dense network that could not be obtained using PLM.

#### 3.2.4. Melting Properties

**Figure A5:**
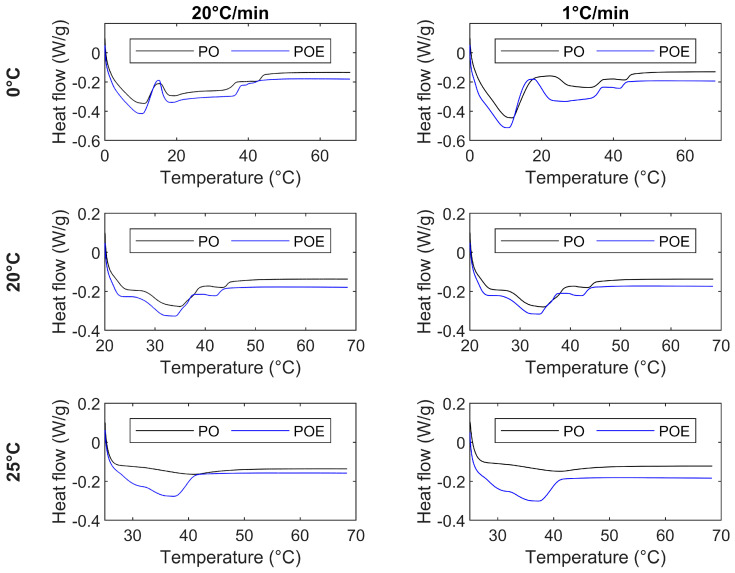
Thermograms obtained using differential scanning calorimetry (DSC) for comparison of PO and POE melting upon application of the different cooling protocols and the subsequent one hour of isothermal crystallization.

**Figure A6:**
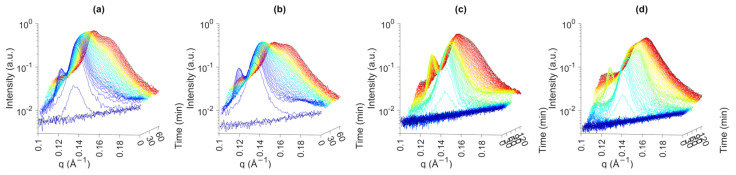
Small-angle X-ray scattering (SAXS) profiles for time-resolved crystallization at 0 °C. (**a**) PO-FC0, (**b**) POE-FC0, (**c**) PO-SC0 and (**d**) POE-SC0.

**Figure A7:**
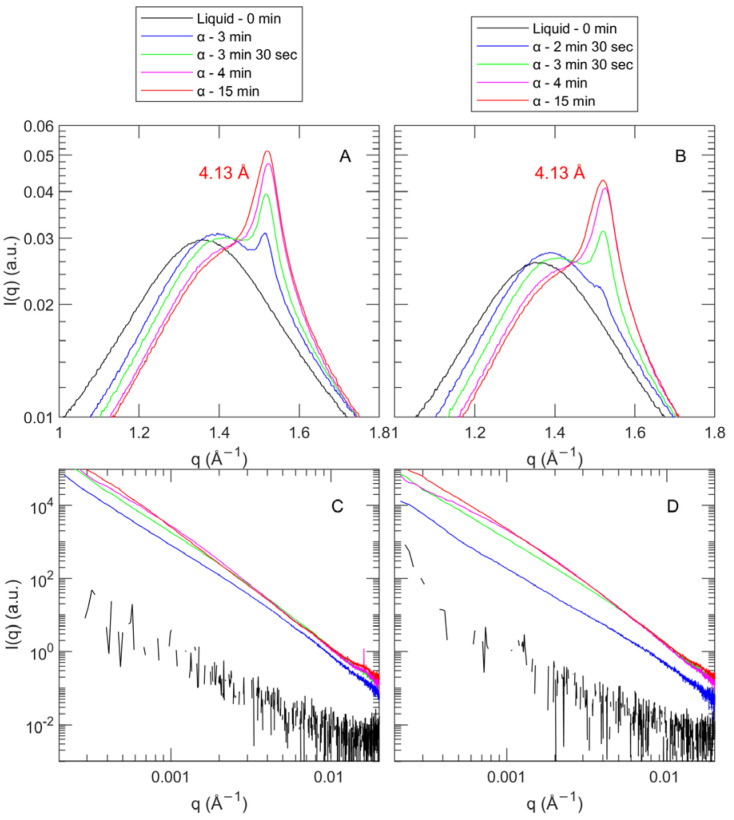
Selected wide- and ultra-small-angle X-ray scattering (WAXS and USAXS) profiles of PO (**A**,**C**) and POE (**B**,**D**) crystallized during FC0 crystallization protocol.

**Figure A8:**
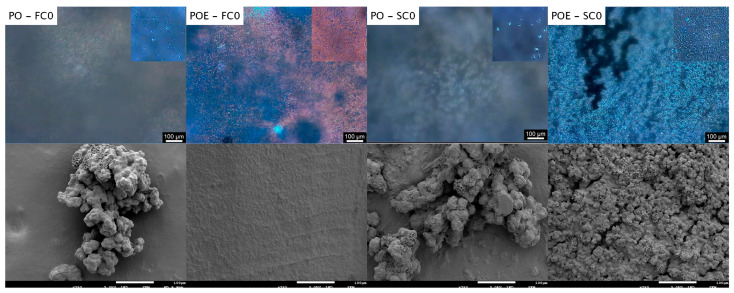
Polarized light microscopy images (PLM) (**upper row**) and cryo-SEM images (**lower row**) of PO and POE, crystallized following FC0 and SC0. The main PLM picture shows the network after 1 h of crystallization. The inset shows the formation of the crystallites/flocs in an early stage. The scale bar represents 100 µm for both techniques.

Appendix B (Figure A5 and Table A5) gives an overview of the melting behaviors of the different samples and cooling protocols. For the samples crystallized at 0 °C, it is clear that melting is directed toward slightly lower temperatures upon the addition of the SE, especially for the slowly cooled sample (SC0), where the effect on the high melting fraction is more pronounced than on the low melting fraction. A similar limited difference was found by Domingues et al. (2022) [38]. Nonetheless, the presence of the two melting fractions, one around 10 °C and a broad one roughly between 20 °C and 40 °C, was maintained.

In contrast to the samples crystallized at 0 °C, the samples crystallized at 20 °C contained a broad melting spectrum between 20 °C and 45 °C. The shape of the melting profile remained unchanged, but the melting temperatures slightly decreased for POE. The most drastic changes were found for crystallization at 25 °C. Both for FC25 and SC25, PO had a peak melting temperature of around 40 °C. For POE, a large fraction had already melted at 30 °C, and melting was almost finished at around 40 °C, indicating that the fat crystal network formed for POE was less heat stable than the one of pure PO. The melting profile, as obtained using DSC, could be affected by many factors. The PLM and cryo-SEM analyses have revealed large morphology and size differences in the fat crystal flocs (microscale). In addition, USAXS has proven that substantial differences in CNP sizes exist.

Overall, in comparison to pure PO, the POE melting profiles do not show any extra fractions in the high melting range. As such, it might be assumed that the SE does not form large, separate crystal structures. Another possibility is that the low concentration of the SE was not detected using DSC.

## 4. Conclusions

In this study, the self-organizing properties of SP-30 with an HLB of 6 were researched in the range of 20–70 °C. It was shown that the different fractions present, ranging from mono- to tetra-substituted stearate and palmitate residues, behave differently during crystallization and melting. At 70 °C, the ester was found to be molten using DSC, PLM, and WAXS, while two SAXS peaks were still prevalent, showing the mesophase liquid crystal behavior. The phase could be described as smectic lamellar.

The isotropic liquid phase was not encountered in the studied temperature range and must thus be situated at >70 °C. Upon cooling, crystallization was encountered around 60 °C. WAXS revealed a hexagonal packing. In SAXS, different crystallization lamellar phases were found and attributed to a complex interplay of mono-, di- and higher ester fractions.

Secondly, it was shown that the addition of 0.5 wt% of SE with an HLB of 6 to palm oil impacted the fat crystal network at different length scales. It was found, using DSC, that the onset of crystallization happened sooner upon the addition of the SE, indicative of a seeding effect. Moreover, the crystallization of different fractions and polymorphic transitions occurred sooner. With WAXS and SAXS, it was shown that, on the nanoscale, the α-2L-to-β’-2L transition happened with and without the presence of the SE, but the transitions happened earlier and were fulfilled faster, as confirmed by narrower crystallization peaks in the DSC analysis.

On the mesoscale, the Scherrer and BWA analyses revealed that the low amount of ester addition decreased the CNP thickness, mainly in samples cooled at a high cooling rate. A Williamson–Hall analysis confirmed that these results could not be directly related to the presence of crystal strain. The USAXS analysis performed in this work showed that under all studied conditions, POE CNPs are smaller than PO CNPs, which was confirmed using cryo-SEM. The POE CNPs seem to reach a limiting size. Moreover, the applicability of USAXS for low and high solid fat samples was illustrated.

On the microscale, the PLM images showed the clear formation of denser and finer networks for POE samples compared to PO, implying the cocrystallization of PO and SE. The optimization of the cryo-SEM de-oiling method allowed us to visualize three-dimensional fat crystal flocs and networks. The results were in good correlation with the PLM images. For pure PO, similar results were obtained as in our previous study, confirming the applicability of the method. For POE, separate flocs were rarely visible, confirming the formation of a finer and more dense network, which might explain the changes in the melting behavior as seen using DSC.

We believe that this research is a step forward toward the study of the fat crystal network comportment in complex food matrices and toward creating functionality-driven fat crystal networks on-demand.

## Figures and Tables

**Table 1:** Overview of parameters obtained from SAXS analysis for PO and POE crystallized under the conditions FC20, SC20, FC25 and SC25. Superscripts indicate a significant change from bigger to smaller. Superscripts a–c indicate significant differences (*p* < 0.05) among different cooling protocols for the same sample, while superscripts A and B indicate significant differences between the two samples.

Sample	Cooling Protocol	SAXS			
		Polymorph and Chain-Length Structure	d_001_ (Å)	Scherrer Average Number of Lamellae (-)	BWA Average Number of Lamellae (-)
PO	FC20	β′-2L	42.1 ± 0.1 ^a,B^	12.0 ± 0.1 ^b,A^	10.1 ± 0.1 ^a,A^
	SC20	β′-2L	42.1 ± 0.1 ^a,A^	12.3 ± 0.1 ^b,A^	10.2 ± 0.1 ^a,A^
	FC25	β′-2L	42.1 ± 0.1 ^a,A^	12.7 ± 0.2 ^a,A^	10.6 ± 0.3 ^a,A^
	SC25	β′-2L	42.1 ± 0.1 ^a,A^	11.5 ± 0.5 ^b,A^	9.6 ± 0.5 ^a,A^
POE	FC20	β′-2L	42.2 ± 0.1 ^a,A^	9.6 ± 0.2 ^b,A^	8.2 ± 0.1 ^bc,A^
	SC20	β′-2L	41.9 ± 0.1 ^b,B^	12.2 ± 0.2 ^a,A^	10.0 ± 0.1 ^ab,A^
	FC25	β′-2L	41.9 ± 0.1 ^b,B^	10.0 ± 0.1 ^ab,A^	8.4 ± 0.1 ^b,A^
	SC25	β′-2L	41.9 ± 0.1 ^b,B^	12.3 ± 0.2 ^a,A^	10.3 ± 0.1 ^a,A^

**Table 2:** Overview of parameters obtained from ultra-small-angle X-ray (USAXS) analysis for PO and POE under the different crystallization conditions studied. * These values were not obtained after 1 h of isothermal time; see main text for discussion.

Sample	Crystallization Protocol	USAXS		
		Slope at Low q (-)	Cut-Off Point (Å^−1^)	Slope at High q (-)
PO	FC0 *	2.7	0.0054	3.4
	FC20	1.9	0.0051	3.5
	FC25	2.2	0.0025	3.4
	SC20	2.0	0.0035	3.3
	SC25	2.2	0.0025	3.4
POE	FC0 *	2.6	0.0066	3.1
	FC20	2.1	0.0070	3.7
	FC25	1.9	0.0068	3.4
	SC20	2.0	0.0067	3.5

**Figure A1:**
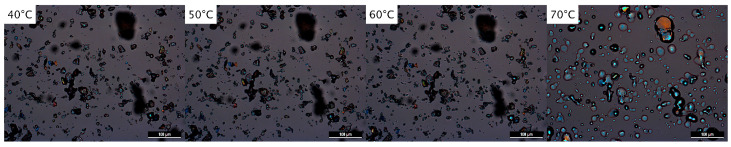
Hot stage melting test of the sucrose ester. Images taken after 10 min of isothermal time at the indicated temperature. Scale bar = 100 µm.

## Data Availability

The data presented in this study are openly available in Zenodo at 10.5281/zenodo.10879185. Raw USAXS and WAXS data are available via the European Synchrotron Radiation Facility: 10.15151/ESRF-DC-1469057171 and 10.15151/ESRF-DC-1576276984.

## Appendix A. Crystallization and Melting of SP-30

**Table A1:** Overview of parameters obtained from differential scanning calorimetry analysis (DSC) during crystallization of the SE SP-30. Superscripts a–c indicate significant differences (*p* < 0.05) among different cooling protocols.

Crystallization Protocol	Onset (°C)	Peak 1 (°C)	Peak 2 (°C)	Peak 3 (°C)	Peak 4 (°C)
FC0	58.9 ± 0.2 ^c^	56.7 ± 0.6 ^b^	39.5 ± 0.2 ^b^	31.4 ± 0.8 ^b^	26.7 ± 1.4 ^ab^
FC20	59.1 ± 0.2 ^bc^	56.9 ± 0.3 ^b^	40.0 ± 0.2 ^ab^	31.7 ± 0.6 ^ab^	27.4 ± 0.6 ^ab^
FC25	59.0 ± 0.1 ^c^	57.1 ± 0.0 ^ab^	39.2 ± 0.5 ^b^	31.2 ± 0.5 ^b^	29.0 ± 0.3 ^a^
SC0	60.9 ± 0.1 ^a^	59.7 ± 0.3 ^a^	42.9 ± 0.3 ^a^	34.2 ± 0.6 ^a^	28.7 ± 0.6 ^a^
SC20	60.8 ± 0.1 ^abc^	59.9 ± 0.1 ^a^	43.3 ± 0.9 ^a^	34.4 ± 1.0 ^a^	25.9 ± 1.0 ^b^
SC25	60.8 ± 0.1 ^ab^	59.9 ± 0.2 ^a^	43.0 ± 0.3 ^a^	34.3 ± 0.4 ^a^	28.8 ± 1.3 ^a^

**Table A2:** Overview of parameters obtained from differential scanning calorimetry analysis (DSC) during melting of the SE SP-30. Superscripts a and b indicate significant differences (*p* < 0.05) among different cooling protocols.

Crystallization Protocol	Peak 1 (°C)	Peak 2 (°C)	Peak 3 (°C)
FC0	44.6 ± 0.1 ^ab^	49.3 ± 0.2 ^ab^	64.8 ± 0.2 ^a^
FC20	44.4 ± 0.1 ^b^	49.2 ± 0.2 ^b^	64.7 ± 0.1 ^a^
FC25	44.5 ± 0.1 ^b^	49.2 ± 0.1 ^b^	64.9 ± 0.2 ^a^
SC0	44.9 ± 0.1 ^a^	49.7 ± 0.1 ^a^	65.6 ± 0.2 ^a^
SC20	44.9 ± 0.2 ^a^	49.7 ± 0.1 ^a^	65.2 ± 0.5 ^a^
SC25	45.0 ± 0.2 ^a^	49.8 ± 0.2 ^a^	65.4 ± 0.4 ^a^

**Table A3:** Overview of the wide- (WAXS) and small-angle X-ray scattering (SAXS) peak positions obtained at various temperatures (see Figure 1).

Temperature (°C)	WAXS		SAXS					
			Peak 1		Peak 2		Peak 3	
	q (Å^−1^)	d (Å)	q (Å^−1^)	d (Å)	q (Å^−1^)	d (Å)	q (Å^−1^)	d (Å)
40	1.50	4.19	0.119	52.8	0.175	35.9	0.352	17.8
50	1.49	4.22	0.118	53.2	0.177	35.5	0.355	17.7
60	Amorphous + 1.46	Amorphous + 4.30	0.118	53.2	0.142	44.2	0.152	41.3
70	Amorphous	Amorphous	0.143	43.9	0.160	39.3	-	-
20	1.48	4.24	0.124	50.7	0.142	44.2	-	-

## Appendix B. Crystallization and Melting of PO and POE

**Table A4:** Overview of parameters obtained from differential scanning analysis (DSC) during crystallization of PO and POE following different protocols. ND = not detected. Time was calculated from the beginning of the cooling period. Superscripts indicate a significant change from bigger to smaller. Superscripts a–c indicate significant differences (*p* < 0.05) among different cooling protocols for the same sample, while superscripts A and B indicate significant differences between the two samples.

Sample	Protocol	Onset		Peak 1		Peak 2		Peak 3	
		Temperature (°C)	Time (min)	Temperature (°C)	Time (min)	Temperature (°C)	Time (min)	Temperature (°C)	Time (min)
PO	FC0	17.5 ± 0.2 ^b,A^	2.8 ± 0.1 ^c,A^	13.8 ± 0.1	3.0 ± 0.1	2.3 ± 0.1	3.6 ± 0.1	0.0 ± 0.1	29.4 ± 0.6
	SC0	21.9 ± 0.5 ^ab,A^	48.2 ± 0.5 ^ab,A^	19.5 ± 0.1	50.8 ± 0.1	1.7 ± 0.1	68.6 ± 0.1	0.0 ± 0.1	93.5 ± 2.0
	FC20 SC20	20.1 ± 0.1 ^b,B^	3.4 ± 0.1 ^bc,A^	20.1 ± 0.1	3.9 ± 0.1	20.0 ± 0.1	14.1 ± 0.5	-	-
		22.3 ± 1.1 ^ab,A^	47.8 ± 1.1 ^ab,A^	20.1 ± 0.1	50.1 ± 0.1	20.0 ± 0.1	60.6 ± 0.2	-	-
	FC25 SC25	25.0 ± 0.1 ^a,A^	30.1 ± 4.6 ^bc,A^	ND	ND	ND	ND	ND	ND
		25.0 ± 0.1 ^a,A^	77.9 ± 6.2 ^a,A^	ND	ND	ND	ND	ND	ND
POE	FC0 SC0	20.6 ± 0.4 ^b,A^	2.6 ± 0.1 ^c,B^	18.6 ± 0.4	2.8 ± 0.1	2.4 ± 0.1	3.6 ± 0.1	0.0 ± 0.1	12.0 ± 2.0
		22.9 ± 0.2 ^ab,A^	47.2 ± 0.2 ^ab,A^	22.5 ± 0.1	47.8 ± 0.1	4.7 ± 0.3 0.0 ± 0.1	65.5 ± 0.3 70.0 ± 0.1	0.0 ± 0.1	80.5 ± 0.7
	FC20 SC20	21.0 ± 0.1 ^b,A^	2.7 ± 0 ^bc,B^	20.8 ± 0.1	2.8 ± 0.1	20.0 ± 0.1	11.8 ± 0.2	-	-
		23.0 ± 0.3 ^ab,A^	47.2 ± 0.3 ^ab,A^	22.4 ± 0.1	47.8 ± 0.1	20.0 ± 0.1	59.3 ± 0.6	-	-
	FC25 SC25	25.0 ± 0.1 ^a,A^	28.8 ± 6.4 ^bc,A^	25.0 ± 0.1	49.6 ± 9.1	-	-	-	-
		25.0 ± 0.1 ^a,A^	70.9 ± 5.3 ^a,A^	25.0 ± 0.1	92.7 ± 8.1	-	-	-	-

**Table A5:** Overview of parameters obtained from differential scanning calorimetry analysis (DSC) during melting of PO and POE at 5 °C/min.

Sample	Protocol	Peak 1 (°C)	Peak 2 (°C)	Peak 3 (°C)	Peak 4 (°C)
PO	FC0	10.7 ± 0.1	19.2 ± 0.1	33.5 ± 0.6	42.6 ± 0.2
	SC0	11.6 ± 0.1	27.6 ± 0.4	34.1 ± 0.3	43.1 ± 0.1
	FC20 SC20	24.3 ± 0.2	34.7 ± 0.2	43.4 ± 0.1	-
		24.4 ± 0.2	34.6 ± 0.1	43.4 ± 0.1	-
	FC25 SC25	41.0 ± 0.1	-	-	-
		41.2 ± 0.1	-	-	-
POE	FC0 SC0	10.3 ± 0.1	18.5 ± 0.8	36.2 ± 0.2	39.3 ± 0.1
		10.7 ± 0.2	26.8 ± 0.4	41.7 ± 0.1	41.7 ± 0.2
	FC20 SC20	23.2 ± 0.2	34.0 ± 0.3	41.7 ± 0.3	-
		23.5 ± 0.2	33.7 ± 0.1	42.1 ± 0.1	-
	FC25 SC25	29.5 ± 2.2	37.6 ± 0.6	-	-
		31.4 ± 0.9	37.2 ± 0.4	-	-

## Appendix D. Crystal Strain Assessments of PO and POE

**Table A6:** Values obtained for the crystal strain (ε) according to the Williamson–Hall plotting method for samples crystallized at 20 °C (samples with highest solid fat content). Superscript a indicates no significant differences (*p* < 0.05) among different cooling protocols for the same sample, while superscript A indicates no significant differences between the two samples.

Sample	Cooling Protocol	Strain ε
PO	FC20	7.2 × 10^−5^ ± 9.8 × 10^−6 a,A^
	SC20	6.1 × 10^−5^ ± 5.3 × 10^−6 a,A^
POE	FC20	6.1 × 10^−5^ ± 7.4 × 10^−6 a,A^
	SC20	4.7 × 10^−5^ ± 8.1 × 10^−6 a,A^

Note: The authors would like to stress that applying the Williamson–Hall technique to a fat crystal network and its accompanying SAXS profile is not ideal. The SAXS profile only shows a clear first-order (001) peak; the second-order (002) peak is often absent due to overlap with the liquid fat contribution; the third-order (003) peak is low in intensity. As such, the Williamson–Hall analysis consists of a line fitted through only two points, which is not ideal. Other authors include WAXS peaks; however, we believe that the q distance between the WAXS and SAXS peaks would distort the analysis even more [45].
